# Experienced Mindfulness Meditators Exhibit Higher Parietal-Occipital EEG Gamma Activity during NREM Sleep

**DOI:** 10.1371/journal.pone.0073417

**Published:** 2013-08-28

**Authors:** Fabio Ferrarelli, Richard Smith, Daniela Dentico, Brady A. Riedner, Corinna Zennig, Ruth M. Benca, Antoine Lutz, Richard J. Davidson, Giulio Tononi

**Affiliations:** 1 Department of Psychiatry, University of Wisconsin-Madison, Madison, Wisconsin, United States of America; 2 Waisman Center for Brain Imaging and Behavior, University of Wisconsin-Madison, Madison, Wisconsin, United States of America; 3 Department of Psychology, University of Wisconsin-Madison, Madison, Wisconsin, United States of America; 4 Lyon Neuroscience Research Center, Lyon 1 University, Lyon, France; Biomedical Imaging Lab, Agency for Science, Singapore

## Abstract

Over the past several years meditation practice has gained increasing attention as a non-pharmacological intervention to provide health related benefits, from promoting general wellness to alleviating the symptoms of a variety of medical conditions. However, the effects of meditation training on brain activity still need to be fully characterized. Sleep provides a unique approach to explore the meditation-related plastic changes in brain function. In this study we performed sleep high-density electroencephalographic (hdEEG) recordings in long-term meditators (LTM) of Buddhist meditation practices (approximately 8700 mean hours of life practice) and meditation naive individuals. We found that LTM had increased parietal-occipital EEG gamma power during NREM sleep. This increase was specific for the gamma range (25–40 Hz), was not related to the level of spontaneous arousal during NREM and was positively correlated with the length of lifetime daily meditation practice. Altogether, these findings indicate that meditation practice produces measurable changes in spontaneous brain activity, and suggest that EEG gamma activity during sleep represents a sensitive measure of the long-lasting, plastic effects of meditative training on brain function.

## Introduction

Meditation can be conceptualized as a set of regulatory and self-inquiry mental training regimes cultivated for various ends, including the training of well-being and psychological health [1], [2],[3]. Accumulating evidence suggests that meditation training induces functional and anatomical neuronal changes [1],[2], [3]. These neuroplastic changes are linked to changes in behavior during cognitive and affective tasks [4],[3]. The investigation of spontaneous brain activity, at rest or during practice, is a sensitive approach to identify these neuroplastic changes. Some studies found meditation-related increases in alpha and theta frequency bands, particularly in frontal midline and prefrontal areas, which were associated with increased relaxation (reviewed in [5]). Another study showed that long-term meditators (LTM) had high-amplitude gamma band (25–40 Hz) oscillations during mental practice localized to lateral frontal and posterior parietal electrodes bilaterally [6]. A gamma power increase in a parietal-occipital region was also reported in another group of LTM during Vipassana meditation [7], whereas in a recent EEG study meditation experts showed higher parietal-occipital gamma power compared to novices during resting wakefulness preceding meditation practice [8].

An important question raised by these studies is whether enhanced EEG activity is state dependent (e.g.– occurring only during meditation and immediately after it), or trait dependent (e.g. – occurring outside of formal meditation practice) [5]. The latter would suggests that long-term meditation training causes lasting neuro-plastic changes in cortico-thalamic circuits, which should be detected in the spontaneous brain activity [9]. This question is difficult to address because meditation training occurs at multiple time-scales. For example it can happen intensively, as during meditation retreats, or less intensively but across a longer period, as during daily practice. Training also occurs, in different contexts, either during formal practice, or when the practitioner intentionally cultivates meditative qualities within her/his daily activities. Another critical methodological issue is that resting state in meditation experts may be an elusive concept. Indeed, it is possible that during wakefulness a meditator may spontaneously enter a meditative state while "at rest" as a consequence of his/her level of training. However, during sleep brain activity is directed by neither conscious effort nor attention, but rather reflects the intrinsic function of cortico-cortical and cortico-thalamic circuits [10]. Thus, the examination of sleep EEG is an effective means to identify individual trait differences in the brain unconfounded by the effect of meditation on waking mentation. [11].

The recent availability of high-density electroencephalography (hdEEG) has greatly enhanced the spatial resolution of standard EEG recordings, thus allowing for better characterization of local plastic changes [12],[13],[14],[15]. HdEEG studies of sleep rhythms, with good spatial and temporal resolution, are extremely sensitive measures of changes in the activity in corticothalamic circuits. For example, we previously showed that slow wave parameters, such as amplitude and slope, display local changes in the brain in response to learning [16],[17],[12]. Moreover, we have demonstrated that sleep spindle activity is decreased in schizophrenic subjects compared to controls [14]. Finally, gamma power has been shown to increase during NREM sleep in human subjects following declarative learning [18].

Here we performed whole night sleep hdEEG (256-channels) in experienced meditators (LTM) and meditation naive individuals matched for age and sex. Even if this is a correlative and not a causal approach, it paves the way for studies on long term plastic effects of meditation on brain activity in healthy humans.

## Materials and Methods

### Participants

Twenty-nine right-handed long-term meditators (LTM, mean age = 50.7 ± 10.4, 15 female) and a group of twenty-nine meditation naive subjects matched for age and sex were recruited. LTM had a history of daily meditation practice of at least 3 years and had participated in at least 3 one-week intensive retreats. Mean duration of meditation training was 15.6 years (± 7.8, SD). Naive subjects had no previous experience with meditation. All LTM participants were proficient in meditation practices, as taught within the framework of either Theravada or Tibetan Buddhisms. These practices included two attention-based meditations, which we referred to as open monitoring (OM) and focused attention (FA), as well as one compassion/loving kindness meditation referred to as metta meditation [1] (Table 1). Briefly, FA meditation involves directing and sustaining attention on a selected object (e.g., breathing), detecting mind wandering and distractors (e.g., thoughts), as well as disengagement of attention from distractors and shifting of the focus of attention back to the selected object. By contrast, OM meditation has no explicit focus of attention, but rather requires nonreactive meta cognitive monitoring of anything that is experienced, thus replacing the "effortful" selection of an object as primary focus with an "effortless" sustained awareness of the rich features of each experience [1]. The practice of compassion/loving kindness meditation is a form of concentration practice where the practitioner focuses his/her mind on the suffering of oneself or others and then on the wish that the individual(s) in question may be happy and free from suffering. After an initial phone screening to collect the medical and psychiatric history, each subject underwent a thorough in-person screening, which included several questionnaires (see below). Sleep-disordered breathing and sleep-related movement disorders were also established/excluded with in-laboratory polysomnography (see below). All subjects provided written informed consent and were instructed to maintain regular sleep-wake schedules in the week preceding EEG recordings. This study was approved by the Institutional Review Board of the University of Wisconsin-Madison.

**Table 1:** Information about long-term meditators' (LTM) practice history: In our convention, Focused Attention meditation encompasses concentrative practices such as Theravada Jhana, or breath awareness meditation.

Total amount of meditation practice in life (in hours)		Mean, 8126; SD, 6066; Min, 1439; Max, 32613		
		Daily practice (Mean 50.5%, SD 28.6%) vs retreat practice (Mean 49.5%, SD 72.6%)		
**LTM's meditation lineage type (N = 26)**		Theravada (N = 21)		
		Tibetan Buddhism (N = 2)		
		Mixed of the above, or of the above with Zen (N = 3)		
**Meditation practices (%)**	Focused Attention meditation (Mean, 33.0; SD, 22.6)	Open Monitoring meditation (Mean, 51.4; SD, 23.6)	Compassion/loving kindness meditation	Other meditations
			(Mean, 14.0; SD, 10.8)	(Mean, 1.6; SD, 6.1)

Open Monitoring meditation encompasses practices such as Vipassana meditation.

### Study design

All subjects underwent in-laboratory hdEEG polysomnography (PSG) that utilized 256 channel hdEEG (Electrical Geodesics Inc., Eugene, OR), as well as standard sleep monitoring leads, including electrooculogram, sub-mental electromyogram, electrocardiogram, bilateral tibial electromyogram, respiratory inductance plethysmography, pulse oximetry, and a position sensor. Participants arrived at the laboratory between 4:00 and 5:00 p.m. for set-up that took approximately two hours. Baseline EEG and an attention and a fear conditioning task were performed. After 9pm the participants were allowed to sleep undisturbed in the laboratory beginning within one hour of their usual bedtime. Additional measures were collected the next day. In this report we will focus only on the EEG data during sleep.

### Self reported measures

The socioeconomic status (SES) measure (Socioeconomic status was measured with the Hollingshead Index of Social Position, [19]) was administered to assess the level of education. The Quick Inventory of Depressive Symptoms [20] and the Symptom Checklist-90-Revised [21] were collected to exclude current depression and other mental health issues. The cut-off for exclusion was a score <2 for both questionnaires. Additionally, each participant completed validated sleep rating scales, including the Insomnia Severity Index (ISI) [22], the Fatigue Severity Scale (FSS) [23], the Epworth Sleepiness Scale [24], a sleep history questionnaire, and the Stanford Sleepiness Scale [25] to assess for symptoms of common sleep disorders, such as restless legs syndrome and obstructive sleep apnea. Thresholds for exclusion were an ISI >10 and/or an FSS >4 and/or an ESS >9.

### Meditation Practice

LTM had an average of 8762 lifetime hours of meditation practice, ranging from 1,526 to 32,349 total hours. The computation of lifetime hours of practice was based on subjects' reports of their average hours of formal (sitting and walking) meditation practice per week, including time spent on meditation retreats (for details see Table 1).

### Sleep PSG assessment and hdEEG data analysis

Sleep staging, which was based on six mastoid-referenced channels (F3, F4, C3, C4, O1, and O2), a sub-mental electromyogram and an electrooculogram, was performed by a registered polysomnographic technologist in 30-second epochs according to standard criteria [26] using Alice® Sleepware (Philips Respironics, Murrysville, PA) The sleep technician was blind to group assignment. PSG recordings were reviewed by a board certified sleep medicine physician, who was able to confirm the absence of sleep disorders in 26 out of 29 LTM. Two LTM met the clinical criteria for sleep-related movement disorders (periodic limb movement arousal index >10/h), while one had sleep-disordered breathing (apnea–hypopnea index >10/h). As a result, data from these three LTM (as well as from the age- and sex- matched meditation naives) were not further analyzed. All-night sleep hdEEG recordings were collected with vertex-referencing, using a NetAmps 300 amplifier and NetStation software (Electrical Geodesics Inc., Eugene, OR). EEG data were sampled at 500 Hz, and a first-order high-pass filter (Kaiser type, 0.1 Hz) was applied to eliminate the DC shift. Data were then band-pass filtered (1–50 Hz), down-sampled to 128 Hz and average-referenced to the mean power in all channels. Spectral analysis was performed for each channel in six-second epochs (Welch's averaged modified periodogram with a Hamming window). For NREM sleep data, a semi-automatic artifact rejection was conducted to remove six-second sleep epochs which exceeded a threshold based on the mean power at either a low (1–4 Hz) or high (20–30 Hz) frequency band [27],[28]. EEG channels in which artifacts affected most of the recording were excluded. REM sleep epochs were visually analyzed and divided in tonic and phasic (i.e., characterized by rapid eye movements). To eliminate artifacts distinctively observed during REM sleep (i.e., muscle twitches, eye movements, heartbeats), independent component analysis (ICA) was performed [29]. After removal of ICA components, power spectral of tonic and phasic REM epochs was computed. We computed six frequency ranges (delta: 1–4.5 Hz, theta: 4.5–8 Hz, alpha: 8–12 Hz, sigma; 12–15 Hz, beta: 15–25 Hz, and gamma: 25–40 Hz) consistent with previous studies from our lab [30]. In order to determine whether group differences in NREM gamma activity were related to increased muscle tone or variation in saccades, we compared the power in the gamma band in neck EMG and EOG derivations [30] between LTM and meditation naive participants.

Sleep cycles were defined according to the modified criteria [31] of Feinberg and Floyd [32] also consistent with our previous studies [33].

### Statistics

Differences in clinical as well as sleep architecture variables were examined using 2-tailed, unpaired t-tests. Topographical analysis was computed after spatially normalizing each subject's topography (z-score across all channels) within the frequency bands of interest as a means to reduce between-subject variance. Group differences in topographical NREM and REM sleep hdEEG power were assessed with statistical non-parametric mapping (SnPM) using a suprathreshold cluster test to identify significant groups of electrodes after accounting for the multiple comparisons due to the numerous electrodes [34]. Briefly, an appropriate threshold t-value was chosen (t = 2, corresponding to α = 0.05 for the given degrees of freedom) before topographic power maps were randomly shuffled between groups (LTM and meditation naive individuals). The size of the largest cluster above the threshold for each reshuffling was then used to create a cluster size distribution. Given the impracticality of computing all possible combinations (4.96×1014), 50000 unique combinations were run for each comparison in order to approximate the actual cluster distribution. The suprathreshold cluster p-value was then determined by comparison of the true cluster size against the approximate maximal cluster size distribution. Bonferroni correction of the p value was performed to account for the 18 tests deriving from separately evaluating 6 different frequency bands over the course of 3 distinct phases of sleep.

The magnitude of NREM sleep EEG gamma power differences between LTM and meditation naive individuals was assessed with the Cohen's d, a measure of the effect size [35]. We also performed correlation analysis between duration of meditation practice in LTM and NREM sleep EEG gamma power, as well as TST and WASO. Statistical analyses were performed using MATLAB (The MathWorks Inc., Natick, MA) and STATISTICA (StatSoft Inc., Tulsa, OK). Non-parametric statistics were used to assess group differences in EMG, EOG, and global EEG gamma power (Wilcoxon rank sum test), as well as to correlate gamma power with variables of interest (Spearman rank correlation). An outlier subject was excluded from the correlation between EEG gamma power and hours of daily practice in LTM based on a suspected over inflation of daily practice (value above 1.5 interquartile ranges from the 75th percentile). Specifically, this LTM had the least amount of retreat time compared with all the LTMs, but the highest amount of non-retreat hours.

## Results

### Demographics and sleep variables

The LTM and the meditation naive groups were matched for age and sex (see Table 2). Additionally, the groups did not differ in education level, as assessed by the socioeconomic status questionnaire (Table 2). LTM had a significantly reduced total sleep time (TST) as well as an increased wake after sleep onset (WASO) compared to meditation naive subjects (Table 2). These sleep parameters did not show a correlation with daily meditation practice. By contrast, the two groups did not differ in sleep onset latency or in the relative time (% of TST) spent in each sleep stage and did not differ in the number of arousals (Table 2).

**Table 2:** Clinical and sleep variables of subject groups.

	Long Term Meditators	Meditation Naive	
	(N = 26)	(n = 26)	
**Clinical Variables**			**p values**
Age	49.4 ± 10.7	47.0 ± 10.8	n.s
Gender (F/M)	14/12	14/12	n.s.
**Sleep Variables**			
Total sleep time (min)	368.5± 51.5	404.8 ± 25.3	**0.001**
Sleep onset latency**^a^** (min)	11.0± 15.35	9.6 ± 10.7	n.s.
REM onset latency**^b^** (min)	120.56± 63.82	107.83± 47.44	n.s.
WASO**^c^** (min)	74.1± 47.1	43.5 ± 18.4	**0.003**
NREM N1 (%)	11.0± 5.7	9.7 ± 5.4	n.s.
NREM N2 (%)	61.3± 7.6	64.3± 10.0	n.s.
NREM N3 (%)	10.7± 6.8	9.0 ± 9.8	n.s.
REM (%)	17.0± 5.3	17.0 ± 4.9	n.s.

a. Sleep onset latency is defined as the time from the beginning of lights out until the first staged epoch other than wake.

b. REM onset latency is defined as the time from Sleep onset until the first staged REM sleep epoch.

c. WASO = wake after sleep onset.

### LTM had higher NREM sleep gamma power compared to meditation naives in a parietal-occipital brain region

**Figure 1:**
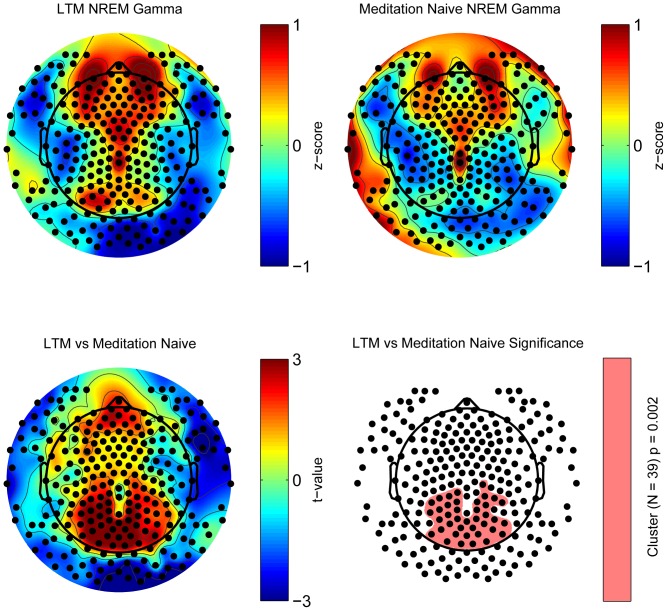
Long-term practitioners (LTM) had higher NREM gamma power (25–40 Hz) compared to meditation naives in a parietal-occipital region. As shown in topographic color plots (colorbar in µV^2^), both groups had maximal EEG gamma power in frontal/prefrontal regions. Furthermore, LTM showed a 35% gamma power increase in a parietal-occipital region compared to meditation naives. The pink area in the white topographic plot depicts the parietal-occipital electrode cluster (N = 39) with a significant power increase in LTM (p = 0.002, Statistical non Parametric Mapping, SnPM).

We first checked for global, frequency non-specific differences, by comparing the absolute average power across the spectrum between the groups. This analysis revealed no difference between LTM and meditation naive individuals (p = 0.094). We then focused on local, frequency specific effects. Whole night NREM sleep EEG power was topographically compared between LTM and meditation naives by spatially normalizing each subject's map to the average power in six frequency ranges (delta: 1–4.5 Hz, theta: 4–8 Hz, alpha: 8–12 Hz, sigma; 12–15 Hz, beta: 15–25 Hz, and gamma: 25–40 Hz). A frequency specific increase in EEG gamma power was found in LTM. Whereas NREM sleep gamma power in both LTM and meditation naives was strongest in frontal/prefrontal areas, and weakest in the temporal regions bilaterally (Figure 1, top row), LTM showed higher relative EEG gamma activity in a parietal-occipital region compared to meditation naives (Figure 1, bottom left). Suprathreshold cluster analysis, a statistical non parametric mapping test (SnPM) which corrects for the multiple comparison problem resulting from the numerous individual electrode tests inherent in hdEEG analysis, confirmed that a parieto-occipital cluster (N = 39 electrodes) showed significantly higher relative gamma power in LTM compared to meditation naive subjects (SnPM, p = 0.002, Figure 1, bottom right). This result survived Bonferroni correction for the 18 tests derived from separately evaluating 6 different frequency bands over the course of 3 distinct phases of sleep (p = 0.05/18 = 0.003). Therefore, we wanted to check whether this topographical increase in gamma power was paralleled by a difference in the non-normalized gamma power between groups. We found a parieto-occipital cluster (N = 46 electrodes) largely overlapping the normalized cluster, that showed significantly higher absolute gamma power in LTM compared to meditation naive subjects (SnPM, p = 0.036). We also examined whether this result was consistent across the night and found similar results in the first three NREM cycles, although the magnitude of the result in the first NREM cycle did not survive multiple comparison corrections after normalization (SnPM, normalized data, cycle 2: N = 20, p = 0.012, cycle 3: N = 30, p = 0.002; absolute data, cycle 1: N = 37, p = 0.0250, cycle 2: N = 44, p = 0.021, cycle 3: N = 54, p = 0.020). Similar results were obtained when breaking down NREM by stages into N2 and N3, with only N2 surviving multiple comparisons corrections after normalization (SnPM, normalized data, N2: N = 23, p = 0.010; absolute data, N2: N = 49, p = 0.019, N3, N = 51, p = 0.014). We suspect that the low values of gamma at the beginning of the night as well as during deep NREM sleep make it difficult to appreciate a difference between groups and therefore the effect is evident but less robust during these times.

We found no gamma power difference in either the electromyographic (EMG) or electrooculographic (EOG) derivations between the two groups (p = 0.589 and p = 0.493 respectively) (Table 2), suggesting that the local gamma increase was not artifactual. SnPM topographic analysis found no significant differences between the two groups for the other five frequency ranges.

### LTM NREM sleep gamma power correlated with meditation training

**Figure 2:**
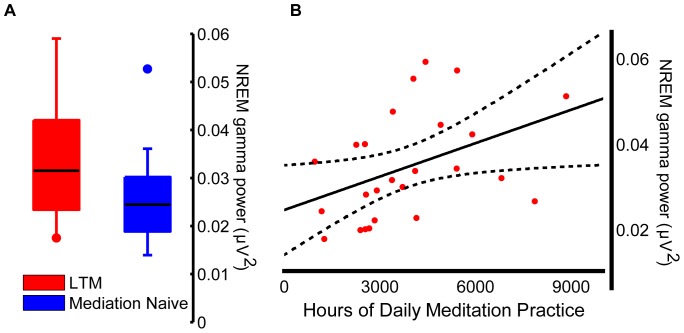
NREM Gamma increase in LTM compared to meditation naives had a large effect size (ES = 0.8, Panel A), and was significantly correlated with the length of meditation daily practice (rho = 0.475, p = 0.017, Panel B).

To further characterize the gamma increase at the individual subject level, we calculated the average NREM gamma power of the parietal-occipital cluster for each subject. The average cluster difference in gamma power between groups was 35% (Figure 2, left panel) and the between group Cohen's d was 0.8, corresponding to a large effect size (more than 50% separation between LTM and meditation naives). We next investigated whether the cluster gamma power was significantly correlated with the overall duration of meditation practice. We found a significant correlation between parietal-occipital NREM gamma power and daily meditation practice, but not retreat time (rho = 0.475, p = 0.017, and rho = 0.029, p = 0.887, for daily practice and retreat time respectively). As gamma power in LTM was correlated with age (rho = 0.487, p = 0.012), we further tested whether the age of meditation naive individuals predicted also EEG gamma power, but no correlation was found (rho = −0.035, p = 0.866). For each group there was also no correlation between gamma power in the cluster and the significantly different sleep architecture variables (TST, rho = −0.195, p = 0.339, rho = 0.287, p = 0.156; WASO, rho = 0.170, p = 0.408, rho = −0.319, p = 0.112, for LTM and meditation naive individuals, respectively). Thus, daily practice was the most sensitive predictor of the correlation with gamma power activity.

### REM sleep gamma power did not differ between LTM and naives

To assess whether the gamma increase of LTM was specific for NREM sleep, topographic EEG analysis of REM sleep was also performed. As previous literature has suggested functional differences between tonic and phasic REM, we separated these two REM sleep patterns in our analysis [36],[37],[38]. Both tonic and phasic REM gamma power peaked in a frontal/prefrontal as well as in a parieto-occipital region in LTM and meditation naives (Figure S1). SNPM analyses revealed no topographical differences in any frequency band in REM sleep between the two groups.

## Discussion

By performing sleep hdEEG (256 channels) recordings in this study we found that LTM had increased parietal-occipital EEG gamma power during NREM sleep compared to meditation naives. This increase was specific for the gamma frequency range, was not found during REM sleep, and was positively correlated with the length of daily meditation practice.

The LTMs recorded in this study were experienced in Focused Attention (FA), Open Monitoring (OM), as well as loving kindness/compassion meditations. An increase in gamma activity was recently demonstrated in expert Buddhist practitioners (>10.000 hours of practice) during a style of meditation which contains features of both loving kindness/compassion and OM meditations [39]. Compared to a group of novices, the expert practitioners showed self-induced, higher-amplitude, sustained EEG gamma-band oscillations, especially over lateral frontal-parietal electrodes, while meditating as well as in the resting state immediately preceding and following meditation [6]. Notably, a link between higher gamma-band activity and stronger cognitive control has been reported by a variety of human electrophysiological techniques, including magnetoencephalography (MEG) [40], scalp EEG [41], and direct cortical recordings [42]. Furthermore, a recent EEG study has found that LTM had increased gamma power in a parietal-occipital area compared to meditation naive individuals during resting state, as well as during meditation practice as compared to baseline [8]. Based on these findings, the authors concluded that enhanced posterior EEG gamma power was a state (meditation-related), and to some same extent trait (resting state associated) feature of meditation practice.

Here we established that LTM had higher EEG gamma power over an extended period of spontaneous brain activity (i.e., whole night NREM sleep) compared to meditation naive individuals. This parietal-occipital gamma increase corresponded to a large effect size (ES = 0.8, allowing more than 50% separation between meditation experts and naives). These findings strongly suggest that changes in EEG gamma activity related to meditation practice are trait (in addition to state) related. Comparing the activity of meditation experts and novices during an unambiguous resting condition is a challenging task [5]. This is because experienced long-term meditation experts are usually able to blend formal meditation session with daily life, and that a meditator might spontaneously generate a meditative state while at rest in the lab out of demand characteristic. In this regard, sleep provides an exquisite window to explore the spontaneous brain activity as well as the function of neural circuits at rest. Brain activity during sleep does not require conscious effort or attention, and a condition of physical immobility is obtained for several hours. Moreover, the recently available combination of standard polysomnography with hdEEG, which provides enhanced spatial and temporal resolution, offers the opportunity to analyze in greater details NREM/REM sleep activity as well as to observe local changes in brain function due to neuroplasticity [43],[16].

Daily practice and meditation retreat could contribute differently to the neuroplastic changes induced by meditation. For instance, meditation frequency (days per week with meditation practice) has recently been shown to reliably predict both higher mindfulness and psychological well-being [44]. In this study we showed that the daily practice, but not the retreat time, predicted the parietal-occipital gamma activity during NREM sleep in LTM. The differential effect of the amount of daily practice and of retreat time on localized NREM gamma activity is to our knowledge the first indication of a specific effect of constant meditation daily practice, but not of intensive retreat practice, on brain neuroplasticity. On one side, this finding raises some methodological issues about how to quantify meditation practice, suggesting the potential usefulness of differentially investigating the contribution of retreat time and daily practice on behavioral and physiological measures. On the other side, it enhances the effectiveness of our approach in revealing stable (trait-like) effects on brain functioning induced by prolonged training during waking.

In the present study LTM had a significantly reduced total sleep time (TST) and increased wake after sleep onset (WASO) compared to meditation naive individuals. A reduction in TST has been recently reported by another sleep study in LTM [45], and it indicates that meditation practice may decrease sleep needs. However other studies [46], [47] investigating sleep architecture didn't find a reduction of TST, suggesting that the sleep architecture is not the most reliable parameter to study the effect of meditation on neuronal plasticity during sleep. Consistent with this idea, we did not find a correlation between the changes in any traditional polysomnographic sleep parameters and meditation practice.

Only a handful of studies so far have investigated the sleep EEG activity of meditation experts beyond sleep architecture. One of these studies explored EEG differences in thirteen individuals with at least 2 years of meditation experience during Transcendental meditation (TM; a form of meditation different from that explored in the current study), resting wakefulness, drowsiness, and sleep and found a progressive slowing of the main EEG frequency from wakefulness to sleep, with no appreciable change in power between wakefulness and meditation EEG [48]. The authors also analyzed the EEG activity of meditation practitioners and of meditation naive control subjects during resting wakefulness, and found no difference in power but a slight slowing in the mean EEG frequency of the practitioners; however, they did not compare the sleep EEG of these two groups [48]. Another study found that eleven long term TM practitioners had increased theta-alpha power during slow wave sleep compared to nine short term practitioners as well as eleven experienced practitioners [49]. Here we found no difference in theta-alpha EEG power between LTM and meditation naive individuals during NREM sleep. Differences in style of meditation practices may account for the discrepancy of these findings. Furthermore, whereas we screened participants for sleep disorders and performed PSG recordings during the first hdEEG night, the LTM recorded by Mason et al. did not undergo such screening. An EEG pattern of alpha wave in delta wave sleep (alpha-delta sleep) is commonly reported in individuals with sleep disturbances, including restless leg syndrome [50] and sleep apnea [51], and is associated with increased arousability and lighter sleep [52]. Notably, their LTM spent a significantly higher amount of time in light (N1) NREM sleep, while we found no difference in N1 between LTM and meditation naive individuals. [45]And finally, the type of meditation practice examined in the current study differs from the practice studied in these other studies.

What is the functional meaning of the gamma increase in LTM that was found here during NREM sleep? A large body of evidence from animal and human recordings have suggested that gamma-frequency activity is implicated during wakefulness in plasticity-related processes, including attention, learning, as well as both working and long-term memory [53]. For instance, an increase in gamma activity occurs when sensory stimuli are attended [54], as well as during the active maintenance of representations during working memory tasks [55],[56]. Several studies employing EEG, MEG and intracranial EEG recordings have also shown that gamma-frequency activity during encoding predicts successful formation of long-term memory [57],[58]. Little is still known about the functional significance of gamma activity during sleep. In this study we found a gamma power increase in meditation experts during NREM sleep in a scalp region overlying posterior parietal and occipital cortical areas. Our finding is in line with the recent report of Valderrama et al., which suggested a functional significance for gamma oscillations during NREM sleep in humans [59]. A possible confound in the study of gamma activity is the presence of muscular or ocular artifacts in the scalp EEG. However, we found no difference in neck EMG and EOG gamma power between LTM and meditation naive individuals. This result therefore strongly indicates that such artifacts do not contribute significantly to our finding.

Previous studies have reported an increase in fast-frequency activity during sleep in pathological conditions such as schizophrenia, depression and insomnia [60],[61],[62]. This increase has been associated with abnormalities in the arousal mechanisms [62]. However, this functional interpretation of gamma activity is not warranted for a number of reasons. First, we did not find a group difference in arousal. Our finding was specific to spatially normalized gamma activity. Furthermore, the topography of the current finding over parietal-occipital electrodes differed from those earlier findings. Finally inter-individual variability in gamma activity among meditators predicted daily meditation experience in life, which has been found to predict positive mental health outcomes [44],[1]. Instead, we speculate here the specific gamma increase is not pathological, but reflects the lasting, plastic effect on specific neuronal circuits of long-term meditation practice. For instance, the parietal cortex has been implicated in directing the focus of attention on a specific object [63], a cognitive skill perfected by attention-based meditation [1]. The finding of higher whole night NREM gamma power in LTM compared to meditation naives could therefore reflect an increase in activity and connectivity within these neuronal circuits due to extensive meditation training. Consistent with this assumption, here we established that NREM sleep gamma band power correlated with the duration of daily meditation practice in LTM. This finding not only confirms a previous report from our group that the amount of EEG gamma power generated during meditation by LTM correlated with the length of their meditation training [6], but also suggests that EEG gamma activity during sleep may represent a trait-like sensitive measure for the neuronal plastic changes determined over time by meditative training.

The increase in NREM sleep parieto-occipital gamma power reported here could also reflect an enhanced activity of the underlying cortical areas in LTM compared to meditation naives. Higher EEG gamma power reflects higher firing rates of the underlying neuronal populations [64], and local changes in gamma oscillations closely mirror underlying activity in both visual [65],[66] and parietal default network-associated cortical regions [67],[68]. During NREM sleep both neuronal firing and gamma power tend to decrease, as does the ability to process sensory information [69],[70]. Thus, a higher gamma activity in LTM could reflect a partially maintained capacity of parieto-occipital sensory and default network-associated areas to process information and maintain some level of awareness, even during a state when usually these cognitive functions are greatly impaired. Consistent with this idea, a higher incidence of dream reports has been found in meditation experts compared to meditation naives even during the deepest stages of NREM sleep [71]. If experienced meditators retain a higher capacity for internal information processing and awareness during NREM sleep compared to meditation naives, such advantage should be reduced during REM sleep, when these functions are partially restored and spontaneous neuronal firing/gamma activity is enhanced compared to NREM sleep [72]. In this study we found that LTM had only a slight, non significant increase in REM sleep EEG gamma power compared to meditation naives in the same parietal-occipital regions (Figure S1).

Limitations of the study include a lack of an adaptation night, which could account for the truncated sleep time (< 7 hrs) in all participants, but it is unlikely to explain the observed group difference in the sleep EEG. Future work will also need to address some of the questions left unanswered in the present study. For example, the relationship found here between higher EEG gamma activity and longer meditation daily practice suggests that gamma power is a good correlate of meditation training. This correlation should be confirmed in longitudinal studies performing EEG recordings in meditation naive individuals before and after meditation training, ideally using only one style of meditation practice. It will also be important to investigate whether the observed gamma increase may be affected by pre-existing "baseline" gamma activity differences between groups (i.e., meditation experts and naives), as previously suggested [6]. Gamma activity has been shown to be influenced by several factors, including age [73], sex [74], and cognitive abilities [75]. However, these factors are unlikely to have contributed to the present findings, given that LTM and meditation naive subjects were matched for age, sex, and did not differ in education level. Future studies should investigate whether the group difference in NREM gamma activity in meditators is associated to a specific meditation practice (e.g. mindfulness meditation vs. compassion meditation) or style of meditation training (e.g. Tibetan Buddhism vs. Theravada Buddhism). Specifically, investigating the acute effect of an intense meditation session in LTM on sleep EEG patterns could help in establishing a causal relationship between meditation training and specific changes in EEG activity. Finally, future experiments combining fMRI with simultaneous hdEEG will be critical to fully characterize the cortical (and possibly sub-cortical) networks underlying the enhanced NREM sleep EEG gamma activity found in this study in meditation experts, whereas studies investigating the healing effects of meditation interventions could explore the ability of EEG gamma power to predict a beneficial effect of such interventions. This work would contribute to identify the neural circuits underlying the EEG correlates of meditation training. It will also help to establish whether EEG gamma activity represents a sensitive and objective measure of the effects of meditative practice on brain function in both healthy subjects and brain disordered patients.

## Supporting Information

Figure S1 **REM tonic as well as phasic gamma power did not differ between LTM and meditation naives.** Topographic color plots showed maximal REM tonic as well as phasic gamma power in frontal/prefrontal regions in both groups. Compared to meditation naives, LTM had a slightly higher power in the same parieto-occipital region significantly more active during NREM sleep, which however failed to reach significance in both tonic and phasic REM (white topographic plots, p = 0.975, and p = 0.810, SnPM). Only the tonic REM topographies are shown.
(TIF)
