# Supplementary figures and images for: Experienced Mindfulness Meditators Exhibit Higher Parietal-Occipital EEG Gamma Activity during NREM Sleep
Source: PLoS One. 2013 Aug 28;8(8):e73417. doi: 10.1371/journal.pone.0073417 (PMC3756031; doi:10.1371/journal.pone.0073417)

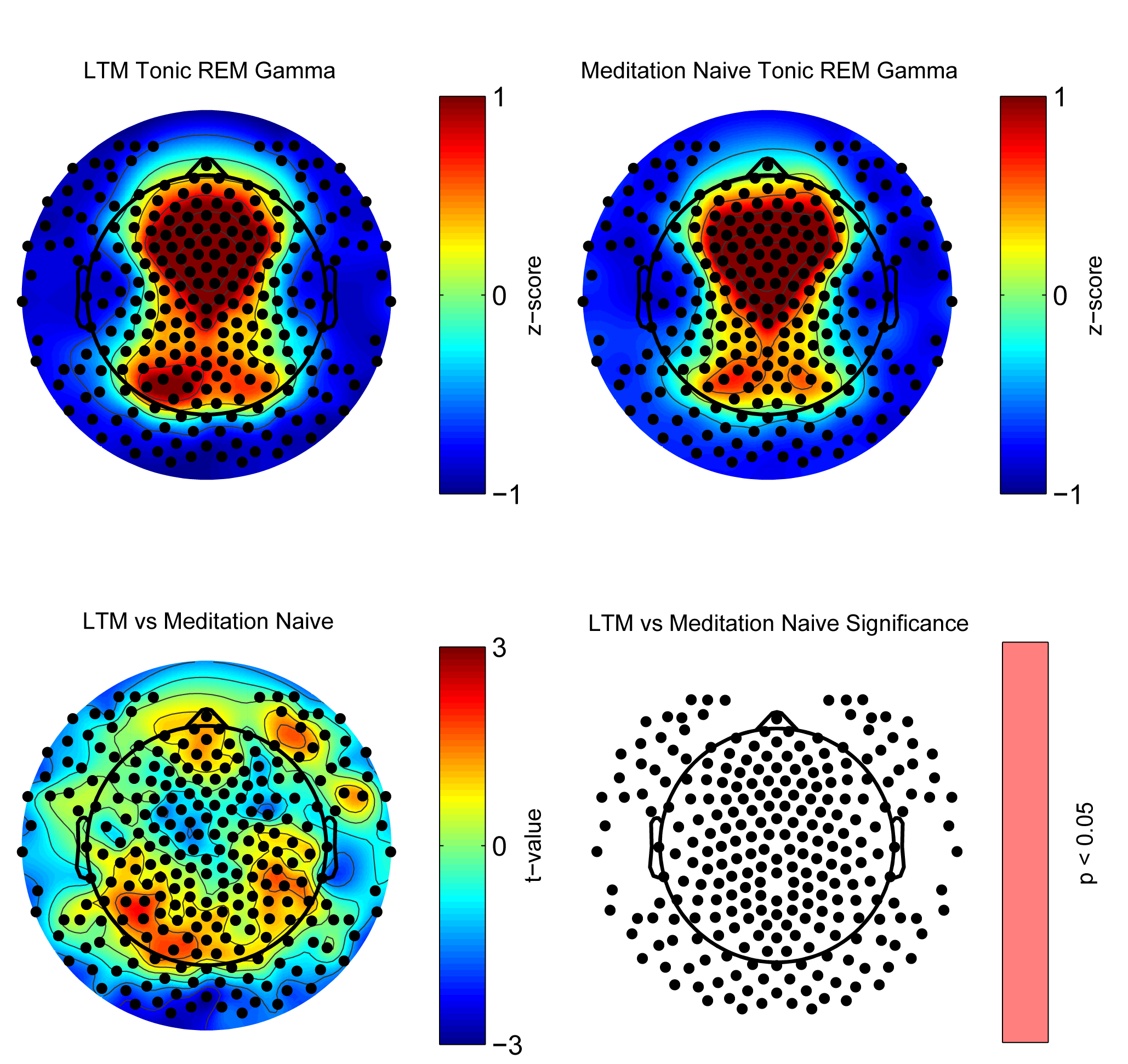

Supplement: Figure S1 — REM tonic as well as phasic gamma power did not differ between LTM and meditation naives. Topographic color plots showed maximal REM tonic as well as phasic gamma power in frontal/prefrontal regions in both groups. Compared to meditation naives, LTM had a slightly higher power in the same parieto-occipital region significantly more active during NREM sleep, which however failed to reach significance in both tonic and phasic REM (white topographic plots, p = 0.975, and p = 0.810, SnPM). Only the tonic REM topographies are shown. (TIF) [file pone.0073417.s001.tif]
